# Cubeb (*Piper cubeba* L.f.): A comprehensive review of its botany, phytochemistry, traditional uses, and pharmacological properties

**DOI:** 10.3389/fnut.2022.1048520

**Published:** 2022-11-22

**Authors:** Badreddine Drissi, Ismail Mahdi, Mouna Yassir, Widad Ben Bakrim, Latifa Bouissane, Mansour Sobeh

**Affiliations:** ^1^AgroBioSciences, Mohammed VI Polytechnic University, Ben-Guerir, Morocco; ^2^African Sustainable Agriculture Research Institute (ASARI), Mohammed VI Polytechnic University, Laayoune, Morocco; ^3^Molecular Chemistry, Materials and Catalysis Laboratory, Faculty of Sciences and Technologies, Sultan Moulay Slimane University, Beni-Mellal, Morocco

**Keywords:** cubeb, *Piper cubeba*, phytochemistry, traditional uses, pharmacological activities

## Abstract

*Piper cubeba* L.f. (Piperaceae), known as cubeb, is a popular traditional herbal medicine used for the treatment of many diseases, especially digestive and respiratory disorders. The plant is rich in essential oil, found mainly in fruits, and this makes it economically important. Many traditional utilizations have been also validated from the plant and its isolated compounds owing to their antioxidant, antibacterial, anti-inflammatory and anticancer effects. These biological activities are attributed to the phytochemicals (phenolic compounds, lignans and alkaloids) and the essential oil of the plant. The present work aims to provide an up-to-date review on the traditional uses, phytochemistry and pharmacology of the plant and discusses the future perspectives to promote its valorization for nutritional- and health-promoting effects.

## Introduction

Aromatic and medicinal plants (AMPs) have been used since antiquity as therapeutic and cosmeceutical agents (1). In addition, the vast ethnopharmacological applications of AMPs have inspired the current research to provide and discover new main drugs against various health disorders (2). The genus *Piper* belongs to the family Piperaceae which has more than 700 species. They are both erect and spreading herbs, shrubs, or rarely trees with great economic and medicinal importance (3). *Piper cubeba* is a native plant of Java and Borneo where the appellation of this plant is the Java pepper. It is one of the plants of the folk pepiraceae species that is used as a spice. The plant is cultivated for its berries, which are rich in essential oil (4). Economically, the plant is an important source of its dried berries as they have several applications in perfumes, cosmetics, and food preservatives (5). In Moroccan cuisine, cubeb is popular in savory dishes and pastries such as *markouts* and the famous *Ras el hanout* spice blend (a popular mixture of herbs and spices used throughout the Middle East and North Africa) (6). Cubeb is marketed by a Swiss company as a refreshing agent and used in various products such as chewing gums, alcoholic and soft drinks, sherbets, gelatin confectionery, and toothpaste (6). Cubeb is also used to flavor alcoholic and non-alcoholic drinks such as Bombay Sapphire gin and Pertsovka, a Russian pepper vodka which is prepared from a cubeb infusion (6). Traditionally, *P. cubeba* is used for the treatment of gonorrhea, dysentery, syphilis, abdominal pain, diarrhea, enteritis, and asthmatic diseases (5). The plant possesses also outstanding pharmacological activities. For instance, *P. cubeba’*s essential oil furnished antiparasitic, antimicrobial, and insecticidal activities (7). In addition, different extracts from the plant demonstrated antioxidant, antimicrobial, nephroprotetive, hepatoprotective, and anti-inflammatory activities (8). These biological activities are due to its chemical composition, especially, phenolic acids and flavonoids, that have been detected in *P. cubeba* extracts (9–11). The plant is also a rich source of lignans particularly cubebin, a bioactive compound with a wide range of biological activities such as antimicrobial, anticaner, and neuroprotective, among others (5, 12). Overall, the most reported modes of action by which *P. cubeba* extracts exert its biological activities involve many intracellular targets, among them the regulation of genes expression, inhibition of oxidative stress, induction of apoptosis and quorum sensing inhibition in pathogenic microbes.

The present review aims to collect and collate the available literature on the botany of plant and provide an insight about its chemical composition and diverse biological activities including antioxidant, anti-inflammatory, antibacterial, wound healing, antidiabetic, and renoprotective activities as well as its agricultural applications. It also highlights future perspectives to further maximize the exploitation of the plant in nutraceuticals, cosmeceuticals, and food applications.

## Literature research

**FIGURE 1:**
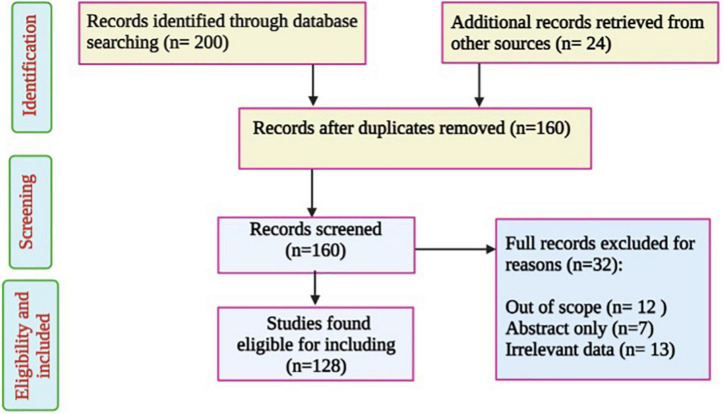
Flowchart of *Piper cubeba* studies inclusion and exclusion criteria.

The literature search was conducted through the Web of science, Scopus, PubMed, SciFinder, and other databases. The keywords used included “*Piper cubeba,”* “chemical composition,” “pharmacological properties,” and “biological activities.” Information has been collected from relevant textbook, reviews, and documents. Duplicated and irrelevant works were excluded as well as non-English documents, and those with unavailable full text (Figure 1). The species name was checked based on the online database.^1^ Various types of information regarding *P. cubeba* are discussed in corresponding parts of the paper.

## Morphological and geographical description

**FIGURE 2:**
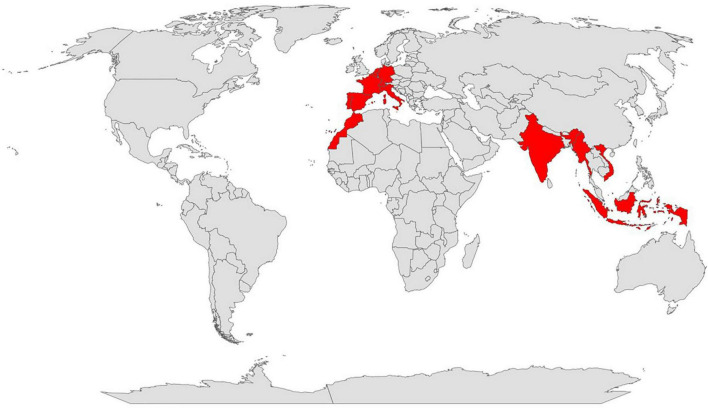
Distribution map of *Piper cubeba*.

**FIGURE 3:**
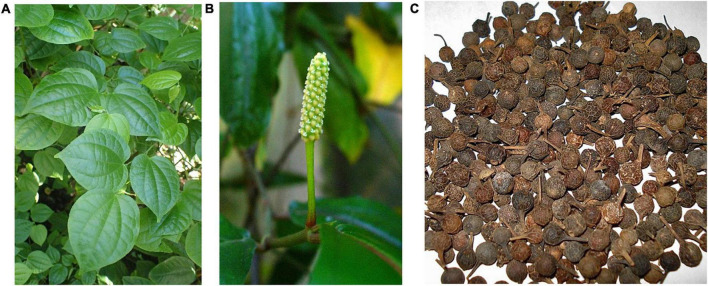
Representative photos of *P. cubeba* **(A)** leaves, **(B)** flowers, and **(C)** berries.

*P. cubeba* is one of the most popular species of the Piperaceae family and the most widespread population of this species is generally found in Indonesia, India, medieval Europe, and North Africa (Figure 2) (5, 13). Cubeb is a woody climbing perennial that has stem and ashy-grey climbing branches. The length is 5–15 m high. The leaves are ovate with cordate or rounded base, glabrous with a thick pedicle, simple, smooth, and pointed at the apex, the lower surface is densely provided with tiny glands embedded. They are completely margined, tough and up to 15 cm long and 6 cm wide (Figure 3A) (5, 9). The flowers are small, dense unisexual that are glued to the peduncles, arranged in 4 cm long scaly spikes that have 2–3 stamens. The female tips have about 50 individual flowers with an ovary of 4 carpels fused with 4 sessile stigmas. Flowering takes place in winter (Figure 3B) (5). The fruits are globose from 6 to 8 mm in diameter. The upper part of the fruit has a diameter of 3–6 mm and covered by grayish brown, pericarp that extends at the base into a straight stem. They have a spicy, aromatic smell and a bitter taste. The fruit has a single dark brown sub-globose seed with a width of 3–4 mm (9) (Figure 3C). The plant has different vernacular names depending on its distribution (Table 1).

**TABLE 1:** Vernacular names of *Piper cubeba* and its distribution.

Vernacular names	Distribution	References
Hab-ul-Urus, Kabâbah, Kebaba, Hhabb El’arûs	Arabic	(6, 104)
Hendkapeghpegh	Armenian	(6)
Kabab Chini; Sital Chini	Bangladesh	(6, 104)
Pimenta-Cubeba (Portuguese)	Brazil	(6)
Gandha Menasu	Canada	(104)
Biji; Bi Cheng Qie, Cheng Qie, Bi Cheng Qie	Chinese	(6, 104)
Cubebe, Cubebepeper, Staartpeper	Dutch	
Tailed Peeper	English	(104)
Cubèbe, Poivre À Queue, Poivre De Java, Poivrier Cubèbe, Quibebes	French	(6)
Tadamiri	Gujrati	(104)
Mahilyun, Karifiyun, Koubeba	Greek	(6, 104)
Kabab Chiniha, Kabab Chini (Bengali), Tadamari (Gujerati), Cubab-Chinee, Kabab Chini, Sheetal Chini (Hindu), Kaba-Chini (Maithili), Vaalmilagu (Malayalam), Kankol (Marathi), Kabachin, Kabab Chini (Oriya), Chinamilagu, Sinamilagu, Valmilagu (Tamil), Chalavamiriyaalu, Tokamiriyalu (Telugu), Kabab Chini (Urdu); Indonesia: Kemukus, Temukus (Java), Rinu Katencar, Rinu Caruluk (Sudanese)	India	
Cubebe, Pepe A Coda	Italian	(6)
Kubeba, Kubebu	Japanese	(6, 104)
Cubebe	Latin	
Valmilaku	Malyalam	
Kubaba	Persian	
Cubeba	Portuguese, Spanish	
Piper De Cubebe	Romanian	(6)
Sungad-muricha	Sanskrit	(104)
Tokamiriyalu	Telugu	
Hind Biberi, Hind Biberi Tohomu, Kebabe, Kebebe, Kebabiye Biber, Kebebiye, Kuyruklu Biber	Turkish	(6)

## Phytochemical composition

*Piper* species are characterized by the production of typical phytochemical compounds such as benzoic acids, amides, chromenos, terpenes, phenylpropanoids, lignans, alkaloids, fatty acids, and hydrocarbons (14). The alkaloid piperine and the two lignans cubebin and hinokinin are the most abundant compounds from the berries (15).

### Lignans

**FIGURE 4:**
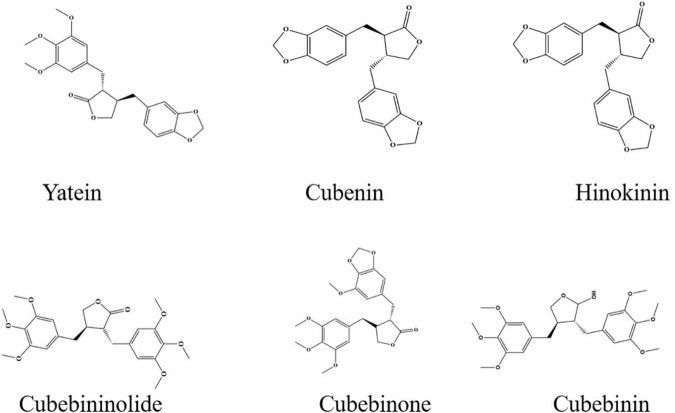
Structure of the most predominant compounds from *P. cubeba.*

Altogether, 28 lignans were annotated from *P. cubeba* (leaves, berries, stalks) using GC, GC-MS, HPLC, and NMR. Out of which, 4 lignans were detected in all plant parts (leaves, berries, stalks), 9 lignans were found only in the leaves and berries, 2 lignans were solely characterized from the leaves and 13 lignans from the berries (Table 2) (5, 16, 17). Cubebininolide, hinokinin, yatein, and isoyatein, which represent the furanofuranic family, are the most known in *Piper* species and were identified in the berries, leaves, and stalks. Ashantin, clusin, cubebin, cubebinone, among others, were identified from the leaves and berries, while the hemiarensin was detected only in leaves (5). Yatein was the most predominant lignan in the berries, more than cubebin, while hinokinin was the most abundant lignan in the leaves and the stem from the Indonesia (5) (Figure 4). Noteworthy, the phytochemical profiling of the plant was mainly annotated from Indonesian flora and most of the studies targeted the extraction and identification of lignans.

**TABLE 2:** Identified lignans from *P. cubeba.*

Compound name	References
Berries, leaves, and stalks	
Cubebininolide	(5, 13)
Hinokinin	
Isoyatein	(5)
Yatein	(5, 16)
Berries and leaves	
Ashantin	(5)
Clusin	(5, 13, 16)
Cubebin	(5, 16)
Cubebinone	(5)
Dihydrocubebin	(5, 13)
α-O-Ethylcubebin	(5)
β-O-Ethylcubebin	
5’-Methoxyhinokinin	
2-(3′,4′-Methylenedioxybenzyl)-3-(3′,4′-dimethoxybenzyl)-butyrolactone	
Di-*O*-methyl thujaplicatin methylether	
Leaves	
Hemiarensin	(5)
Berries	
Cubebinin	(5, 16)
(+)-Dihydroclusin	(16)
(−)-Haplomyrfolin	
(8R,8′R)-4-Hydroxycubebinone	(13)
(8R,8′R,9′S)-5-Methoxyclusin	
R-Asarone	
R-Methylcubebin	
Magnosalin	
(−)-Yatein	
2,4,5-trimethoxyphenylacetone	
Ethoxyclusin	
(−)-Dihydroclusin	
1-(2,4,5-trimethoxyphenyl)-1,2-propanedione	

### Volatile compounds

Altogether, 91 volatile compounds were characterized in the essential oil, oleoresin, ethanol, and dichloromethane extracts from *P. cubeba*. Methyl eugenol (41.31%), eugenol (33.95%), beta-cubebene (18.3%), and alpha-cubebene (4.1%) dominated the essential oil while cubebol (26.1%) and beta-cubebene (12.3%) were the major compounds indentified from the oleoresin. As for ethanol extract, copaene was the most dominant compound that represented 13.47% of the extract followed by napthalene, 1,2,3,5,6,8a-hexahydro with 10.36%. Significant differences were detected between the extracts (Table 3). For instance, α-cubebene was found to be 4.1% in the essential oil content, 3.5% in the oleoresin, and 2.07% in the ethanolic extract; however, it was not detected at the dichloromethane extract. Copaene, another example, was detected only at the ethanol extract with 13.47% (18). Propylene glycol dominated the dichloromethane with 23.82%; however, it was not found in all other extracts (19). These differences might be attributed to the different extraction methods, detection techniques as well as geographic distribution and genetic chemotypes. Noteworthy, further experiments are required to annotate the non-volatile constituents of the extracts such as the ethanol extracts.

**TABLE 3:** Identified compounds from essential oil, oleoresin, ethanol and dichloromethane extracts of *P. cubeba* berries.

Compound name	Relative abundance (%)				References
	Essential oil	Oleoresin	Ethanol extract	Dichloromethane	
α-Phellandrene^b^	0.2	−−−	−−−	−−−	(20, 21)
α-Terpinene^b^	Trace	−−−	−−−	−−−	(21)
α-Caryophyllene^a^	1.14	−−−	0.62	−−−	(18)
α-Copaene^b^	8.8	6.2	−−−	−−−	(18, 21)
α-Cubebene^a,b^	4.1	3.5	2.07	−−−	
α-Guaiene^b^	0.2	0.2	−−−	−−−	(18)
α-Gurjunene^b^	0.3	0.6	−−−	−−−	
α-Humulene^b^	0.9	1.5	−−−	−−−	
α-Muurolene^b^	0.6	1.3	−−−	−−−	
α-Pinene^b^	4.1	0.5	−−−	−−−	(20, 21)
α-Selinene^a^	0.47	−−−	−−−	−−−	
α-Terpineol^a^	0.96	−−−	−−−	−−−	
α-Terpinolene ^a^	1.41, 0.9	−−−	−−−	−−−	
α-Thujene ^b^	4.5	0.6	−−−	−−−	
β-Caryophyllene^a,b^	5.65, 3.7	3.7^b^	−−−	−−−	
β-caryophyllene oxide^a,b^	0.96, 0.3	−−−	−−−	−−−	
β-Caryophyllene, trans^a^	−−−	−−−	−−−	1.9	(19)
β-Cubebene^b^	18.3	12.3	−−−	−−−	(18, 21)
β-Elemene^a,b^	0.66, 0.6	1.4^b^	−−−	−−−	(20, 21)
β-Myrcene ^a,b^	1.23 ^a^, 0.3 ^b,^	−−−	−−−	−−−	
β-Ocimene ^a^	0.30	−−−	−−−	−−−	
β-Ocimene, trans	−−−	0.2	−−−	−−−	(21)
β-Phellandrene^b^	5.9	1.2	−−−	−−−	(20, 21)
β-Pinene^b^	0.7	0–2	−−−	−−−	
γ-Amorphene^b^	2.0	−−−	−−−	−−−	(18)
δ-3-Carene^b^	Trace	−−−	−−−	−−−	(20, 21)
δ-Cadinene^a,b^	0.19^a^, 0.9^b^	2.7	−−−	−−−	(18, 20, 21)
δ-Elemene ^a,b^	0.9	0–6^b^	−−−	−−−	(20, 21)
δ-Terpinene^b^	0.2	−−−	−−−	−−−	(21)
allo-Aromadendrene^b^	3.1	3.5	−−−	−−−	(18)
Aromadendrene^b^	0.1	−−−	−−−	−−−	
Bicyclogermacrene^b^	1.5	0.7	−−−	−−−	
Cadina-1(2),4-diene-trans^b^	0.2	1.0	−−−	−−−	
Camphene^b^	Trace	−−−	−−−	−−−	(20, 21)
Camphor^b^	Trace	−−−	−−−	−−−	(18, 21)
Caryophyllene oxide^a^	−−−	−−−	−−−	0.65	(19)
cis-Sabinene hydrate^b^	0.9	0.3	−−−	−−−	(18, 21)
Citral^a^	−−−	−−−	−−−	0.6	(19)
Citronellol^a^	0.10	−−−	−−−	−−−	(20, 21)
Copaene ^a^	−−−	−−−	13.47	−−−	(18)
Cubebol^b^	4.7	26.1	−−−	−−−	
Cubebol stereoisomer^b^	0.2	5.6	−−−	−−−	
Cyclohexene^a^	−−−	−−−	−−−	0.82	(19)
D-Limonene^a^	0.12	−−−	−−−	−−−	(20, 21)
Estragole^a^	0.15	−−−	−−−	−−−	
Ethynylbenzene^a^	−−−	−−−	−−−	0.71	(19)
Eugenol^a^	33.95	−−−	−−−	−−−	(20, 21)
Germacrene D^a,b^	0.15^a^, 2.6^b^	8.3^b^	−−−	−−−	(18, 20, 21)
Germacrene-B^b^	0.1	−−−	−−−	−−−	(18)
Hexanal^a^	−−−	−−−	−−−	0.38	(19)
Isocaryophyllene ^a^	−−−	−−−	2.23	−−−	(18)
Isocembrol^a^	0.16	−−−	−−−	−−−	(20, 21)
Isoledene^b^	Trace	−−−	−−−	−−−	(18, 21)
Isopropylpyrazine ^a^	−−−	−−−	1.52	−−−	(18)
Ledol ^a,b^	−−−	2.9	6.25	−−−	(18, 21)
Linalool^a,b^	0.22, 4.9	1.5 ^b^	−−−	−−−	(20, 21)
Methyl eugenol^a^	41.31	−−−	−−−	−−−	(20, 21)
Muurola-4(14), 5-diene trans ^b^	−−−	0.4	−−−	−−−	(18)
Naphthalene, 1,2,3,4,4a,5,6,8a-oct ^a^	−−−	−−−	1.83	−−−	
Naphthalene, 1,2,3,4,4a,7-hexahydro ^a^	−−−	−−−	2.23	−−−	
Napthalene, 1,2,3,5,6,8a-hexahydro ^a^	−−−	−−−	10.36	−−−	
p-Cymene^b^	1.0	−−−	−−−	−−−	(21)
*p*-Cymene-8-ol ^a^	3.50	−−−	−−−	−−−	(20, 21)
Phytol	−−−		−−−	0.44	(19)
Propylene glycol	−−−	−−−	−−−	23.82	(19)
Sabinene^b^	19.4	5.8	−−−	−−−	(20, 21)
Spathulenol^a,b^	0.18, 0.4	−−−	−−−	−−−	
Terpinen-4-ol^a,b^	1.80, 0.9	−−−	−−−	−−−	
Terpinolene^b^	Trace	−−−	−−−	−−−	(18, 21)
trans-Sabinene-hydrate^b^	0.5	0.1	−−−	−−−	(18, 21)
Undecane^a^	−−−	−−−	−−−	0.31	(19)
Undecanoic acid, ethyl ester^a^	−−−	−−−	1.36	−−−	(18)
Viridiflorol^a,b^	0.39, 0.3	−−−	−−−	−−−	(20, 21)
Zingiberene^b^	0.1	−−−	−−−	−−−	(18)
(2R,2′′R)-(-)-Tetrahydro-2,2-′-biuranyl-5,5′-dione^a^	−−−	−−−	−−−	1.30	(19)
(2S)-3-Methyl-3-buttene-1,2-diol	−−−	−−−	−−−	0.15	(19)
(E)-Geraniol^a^	0.19	−−−	−−−	−−−	(20, 21)
1,3,6-Heptatriene,2,5,6-trimethyl ^a^	−−−	−−−	4.54	−−−	(18)
1,6-Octadiene^a^	−−−	−−−		0.25	(19)
1,8-Cineole^a,b^	2.94, trace	0.8 ^b^	−−−	−−−	(20, 21)
1H-Cycloprop [e] azulene, decahydro ^a^	−−−	−−−	3.71	−−−	(18)
1-Naphthalenol, decahydro-4a-methyl ^a^	−−−	−−−	1.37	−−−	(18)
2-Pentene^a^	−−−	−−−	−−−	0.5	(19)
3,7-Dimethyl-2,6-octadienal^a^	−−−	−−−	−−−	1.42	
3-Carene^a^	−−−	−−−	−−−	0.18	
3-Cyclohexene-1-methanol	−−−	−−−	−−−	0.22	
4-Hydroxy-4-methyl-2-pentanone^a^	−−−	−−−	−−−	0.29	
4-Methylthiazole ^a^	−−−	−−−	0.18	−−−	(18)
5-Undecyne ^a^	−−−	−−−	1.06	−−−	(18)

Trace: < 0.05; ^a^GC-MS; ^b^MS and −−− (not found).

### Fatty acids and others

Altogether, 19 fatty acids along with their esters were annotated from *P. cubeba* berries. They include dodecanoic acid (lauric acid, 24.05%), hexadecanoic acid (palmitic acid, 11.37%), 9-octadecenoic acid (10.00%), decanoic acid (capric acid, 2.62%), 9,12-octadecadienoic acid (Z,Z) (2.50%), octadecanoic acid (2.08%), methyl decanoate (capric acid methyl ester, 1.80%), tetradecanoic acid (myristic acid, 1.66%), ethyl-(R,E)-4-hydroxy-3-methylpent-2-enoate (1.12%), along with other compounds where their concentrations did not exceed a relative abundance of 1 like palmitic acid methyl ester (0.65%) and octanoic acid (caprylic acid) (0.18%) (19). The dichloromethane fraction gave the presence of two phenolic compounds which are 4-vinylphenol and 2,4-bis(1,1-dimethylethyl)-Phenol (19). Besides, several flavonoids and phenolic acids were isolated from the aqueous extract of *P. cubeba* fruits such as rutin, catechin, gallic acid, caffeic acid, syringic acid, ferulic acid (20, 21).

### Phenolic, flavonoid and minerals contents

Determination of total phenolic contents from piper fruit was investigated using the Folin-Ciocalteu method. It amounted 123.1 and 185.65 μg of GAE/g extract from the ethanolic extract (20, 21), and 1,280 μg of GAE/g extract from the for the methanolic extract (20, 21). The quantification of flavonoids was carried out as well and amounted 65.83 μg QE/g extract from in ethanolic extract (20, 21). *P. cubeba* fruit aqueous extract contained zinc (Zn), selenium (Se), magnesium (Mg), phosphorus (P), iron (Fe), and manganese (Mn) (22).

## Traditional uses of *P. cubeba*

*P. cubeba* is extensively used in several ways as powder, decoction, as an essential oil for numerous purposes. The fruit is frequently used to treat various diseases such as gastro-tonic and abdominal pain, anti-asthmatic, and sedative. In Morocco, the plant has been listed among the medicinal plants used in cancer treatment. Many of these traditional uses were supported by scientific evidence. These include antibacterial, nematocidal, analgesic, and anticancer activities. Moreover, *P. cubeba* fruits are widely exploited in spice market, and also used as food preservative, coloring aid and in cosmetics (9–11).

## Biological and pharmacological activities

Several studies have shown that *P. cubeba* extracts, essential oils, and their constituents are endowed with many biological and pharmacological properties such as antioxidant, anti-inflammatory, antidiabetic, anticancer, reno-hepatoprotective, immunomodulatory, antidepressant, antimicrobial, anti-parasite, insecticidal, wound healing, and antidepressant activities. However, plant constituents can interact with biological components in the cells such as proteins and nucleic acids, toxicity studies are mandatory to ensure the beneficial and biosafety effects of the tested materials. In this section, we address the toxicity of *P. cubeba*, describe the biological and pharmacological activities of its extracts and/or compounds, underline some mechanisms of action, and discuss major findings.

### Toxicity studies

Toxicity assessment of *P. cubeba* extracts were reported in many studies. For instance, it was shown that, using 3-(4,5-dimethylthiazol-2yl)-2,5-diphenyltetrazolium bromide (MTT) assay, *P. cubeba* extracts were not toxic to RAW 264.7 cells (monocyte/macrophage-like cells). In addition, normal oral fibroblasts treated with *P. cubeba* based compounds mainly methylcubebin, dihydrocubebin, and hinokinin showed neither cytotoxicity signs nor morphological changes (12). *In vivo*, it was found that the female Wister rats fed with the methanol extract of *P. cubeba* fruits were safe up to a maximum dose of 2,000 mg/kg body weight. It induced neither changes in behavioral patterns nor signs and symptoms of toxicity nor mortality (23). In another study, (−)-hinokinin, obtained by partial synthesis from (−)-cubebin isolated from the fruits of *P. cubeba*, was orally administered (1 mL/rat) to male Wistar rats daily for 1 week. This treatment did not cause any significant weight or water intake changes during the period of the experiment (24). Elsewhere, male Wistar rats subjected to treatment by *P. cubeba* essential oil ranging from 50 to 3,000 mg/kg showed no mortality nor overall behavioral alteration such as shaking, convulsion, writhing, chewing, pupil size, feeding behavior, and fecal output (25). The biosafety status was also monitored using other *Piper* species such as *P. longum* L. fruits which caused no significant acute (24 h) or chronic (90 days) mortality in mice (26). Likewise, the leaf extract from *P. betle* was nontoxic on the glyoxalase system of Swiss albino mice after 2 weeks of oral administration at 1.5 and 10 mg/kg (27).

### Antioxidant activity

Plant constituents are commonly known as the best source of antioxidants that neutralize reactive oxygen species (ROS) and free radicals (28). Consequently, an increasing attention is given to the antioxidant potential of plant-based molecules and their role in benefiting health and preventing aging and oxidation-related diseases (29). Like other plants, the antioxidant activities of *P. cubeba* extracts and essential oil were widely evaluated (Table 4). Many *in vitro* assays were used, mainly 2,2-diphenyl-1-picrylhydrazil (DPPH) radical scavenging, ferric-reducing antioxidant power (FRAP), β–carotene bleaching, thiobarbituric acid reactive substances (TBARS), phosphomolybdenum, CUPRAC (Cupric reducing antioxidant capacity), and total antioxidant capacity (TAC) assays (Table 4).

**TABLE 4:** Antioxidant activity of *P. cubeba*.

Extract/compound	Used method	Effects	References
**Dry fruits**			
Methanol	DPPH	IC_50_ = 58.75 μg/mL	(50)
**Dry berries**			
n-hexane	DPPH	% inhibition = 46% (at 10 mg/mL)	(105)
Dichloromethane		IC_50_ = 650 μg/mL	
MeOH		IC_50_ = 271 μg/mL	
**Fruits**			
Essential oil	DPPH	IC_50_ = 110.00 ± 0.08 μg/mL	(106)
	FRAP	IC_50_ = 106.00 ± 0.11 μg/mL	
	β-Carotene-linoleate	IC_50_ = 315.00 ± 2.08 μg/mL	
Methanol	DPPH	IC_50_ = 11.3 ± 0.3 μg/mL	(107)
Essential oil		% inhibition = 17.53 ± 0.030%	(108)
Methanol		% inhibition = 66.20 ± 3.20%	(109)
Essential oil		IC_50_ = 78.9 μg/Ml	(8)
Hydro-alcoholic		% inhibition = 20.23% (at 500 μg/mL)	(110)

IC_50_, half maximal inhibitory concentration; DPPH, 2,2-diphenyl-1-picryl-hydrazyl-hydrate; FRAP, ferric-reducing antioxidant power.

The antioxidant capacity of six extracts from *P. cubeba* fruits (petroleum ether, benzene, ethyl acetate, acetone, methanol, and ethanol) were evaluated *in vitro* (18). At 200 μg/mL, it was noticed that the ethanolic extract was the most potent in inhibiting DPPH, followed by acetone and ethyl acetate extracts at the highest concentration tested. Comparable pattern of antioxidant activities between extracts was observed using other methods such FRAP and CUPRAC assays. The antioxidant effect was also reported using *P. cubeba* essential oil. In fact, essential oil at 500 μg/mL elicited a good radical scavenging activity (84%) compared to ascorbic acid (92%), the reference antioxidant compound (25). Similarly, the radical scavenging activity induced by *P. cubeba* essential oil using DPPH and ABTS assays was up to 38.69% higher than the one that was exhibited by the essential oil of *P. nigrum* L. (7). Three other *Piper* species, namely *P. guineense* Schum and Thonn, *P. nigrum* L. and *P. umbellatum* L. showed also endowed scavenging activity (up to 89.9% inhibition) and metal chelating activity (up to 93.9% inhibition) (30). Consequently, *Piper* species can be considered as a great source of modulators of free radical induced disorders.

### Cytotoxicity and anti-cancer activity

As several cancer chemotherapeutics are derived from plant-based molecules (31), multiple studies have explored the antitumor and cytotoxic activity of *P. cubeba* extracts against model cancer cells using several methods, mainly MTT test. For instance, the dichloromethane extract from *P. cubeba* fruits induced apoptosis on triple negative breast cancer cell lines (MCF-7 and MDA-MB-231), colon cancer cells (HT-29), cholangiocarcinoma cells (KKU-M213), with less cytotoxicity against normal fibroblast (L929). Sequential extraction showed that one fraction, named dichloromethane 15 (DE15), significantly enhanced multi-caspases activity in the breast cancer cell line MDA-MB-231 in a time-dependent fashion (19). Similarly, Graidist et al. (32) showed that the methanolic crude extract of *P. cubeba* fruits exhibited a better cytotoxic activity against MDA-MB-468 and MCF-7 breast cancer cell lines than the dichloromethane crude extract. From the six fractions, the most active fraction had an IC_50_ of > 4 μg/mL. DNA fragmentation assay demonstrated an apoptosis pattern in MCF-7, MDA-MB-468, MDA-MB-231, and L929 cancer lines, but not in MCF-12A normal cells (32).

Several compounds extracted from *P. cubeba* have been explored for their anticancer and cytotoxic potential, among them (–)-cubebin and its derivatives. Niwa et al. (33) studied the safety profile of (–)-cubebin by testing its cytotoxicity, mutagenicity, cell proliferation kinetics, and induction of apoptosis in human colon adenocarcinoma cells (HT29). MTT assay showed that (–)-cubebin was cytotoxic at 280 μM, whereas no cytotoxicity was demonstrated below 28 μM. In addition, micronucleus assay revealed that (–)-cubebin was not mutagenic, did not alter cell-growth kinetics over 4 days, and absence of induced apoptosis after 24 h (33). Moreover, the effect of *P. cubeba* extract and its major lignans (cubebin, dihydrocubebin, ethylcubebin, hinokinin, and methylcubebin) were evaluated on the larynx (Hep-2) and oral (SCC-25) squamous carcinoma cells and normal fibroblasts. They all decreased cell proliferation and migration with no change in cellular morphology and no genotoxic effects. This was attributed to the alteration of the expression of genes and proteins involved in the inflammatory process (12). This study concluded that cubebin and methylcubebin isolated from *P. cubeba* had a great effect on the proliferation, migration, and genotoxic profile of the head and neck cancer cells. Next to *P. cubeba*, the ethanolic extract *P. nigrum* L. was toxic to MCF-7 cells likely through calf thymus DNA (CT DNA) intercalation and damage (34). Additionally, compounds from Korean *P. kadsura* A549 such as kadsuketanone A, piperolactam A, and piperolactam B elicited a cytotoxic effect toward the SK-OV-3 (ovarian cancer cells), A549 (non-small cell lung adenocarcinoma), SK-MEL-2 (skin melanoma), and HCT-15 (colon cancer cells) cell lines (35). Many other *Piper* plants were reported to be used traditionally to treat cancer or cancer-like symptoms. These include *P. aduncum* L. for skin tumors (36), *P. longum* L. for breast cancer (37), *P. nigrum* L. for abdominal, respiratory, or gastric tumors/cancers (38–40). A plenty of extracts and compounds from the genus *Piper* were proved to be cytotoxic against cancer cells. For instance, amide alkaloids represent up to 53% of the most bioactive compounds. Outstandingly, piperlongumine showed excellent toxicity against dozens of cancer cell lines both *in vitro* and *in vivo* (41). Hence, conducting clinical anticancer studies on *Piper* plants, among them *P. cubeba*, and their bioactive principles seems to be worthwhile.

### Anti-inflammatory activity

Several disorders and diseases are linked to the inflammatory responses including diabetes mellitus, rheumatoid arthritis, neurodegenerative diseases, and cancer (42). Many anti-inflammatory agents were isolated from plants such as curcumin, quercetin, capsaicin, resveratrol, and epigallocatechin-3-gallate (43). The anti-inflammatory activity of *P. cubeba* was studied in various studies both *in vitro* and *in vivo*. For instance, Mazlan et al. evaluated the anti-inflammatory effect of *P. cubeba* extracts and fractions by monitoring the nitric oxide (NO) production in lipopolysaccharide (LPS) RAW 264.7 cells. Compared to untreated cells, those treated with *P. cubeba* extracts and fractions showed a significant reduction in NO production up to 74.17%. The methanolic extract was the most potent in reducing NO production (44). Similarly, *P. cubeba* methanolic extract decreased NO production in macrophage RAW264.7 and HEK293T cells without any evidence of cell toxicity. In addition, it inhibited the expression level of proinflammatory cytokines such as iNOS and IL-6, downregulated NF-κB activation, and reduced the phosphorylation of IκBα, IKKα/β, Akt, p85, Src, and Syk (45). Interestingly, molecules such as 5-acetyl-2,3-dihydro-7-methyl-1H-pyrrolizine were identified in *P. cubeba* fruits aqueous extract and shown to reduce LPS-induced inflammation and inhibit LDL oxidation (46).

Some mechanisms underlying the inflammatory effect of *P. cubeba*, especially its essential oil, were studied using carrageenan induced pleurisy in rats (25). At 600 mg/kg, the essential oil substantially reduced the paw edema, the weight of cotton pellet granuloma, and the exudate volume. In addition, the level of polymorphonuclear cells was decreased as well as lung tissue myeloperoxidase, NO, and proinflammatory cytokines such as TNFα and IL-1β. The anti-inflammatory effects observed *in vivo* were attributed to the fact that *P. cubeba* essential oil contains sabinene, γ-terpinene, 4-terpineol, and α-thujene, known to be endowed with antioxidant and anti-inflammatory properties. Comparatively, the bioactive n-hexane and methanolic extracts from *P. kadsura* aerial parts were found to contain kadsuketanone A, *ent-*germacra-4(15),5,10(14)-trien-1β-ol, aristolactam A II, *trans*-2,3-diacetoxy-1-[(benzoy1oxy)methyl]-cyclohexa-4,6-diene, and piperolactam A and B. Both extracts induced a significant inhibition of both PGE_2_ and NO production in the LPS-activated microglia cells (35). Thus, expanding the research on the anti-inflammatory potential of *Piper* species can be a promising strategy to develop *Piper-*derived drugs or adjuvant medicines suitable for the treatment of inflammation-related disorders.

### Antidiabetic activity

Diabetes is one of the most prevalent health problems worldwide (47). It has serious health consequences leading to increasing mortality. Synthetic anti-diabetic drugs have unavoidable side effects. Therefore, medicinal plants and their active components can act as alternative anti-diabetic medicines. Many plants are renowned for antidiabetic potential including *P. cubeba* (47–49). Noteworthy, the role of *P.cubeba* in the management of diabetes has been underexploited and is yet to receive sound scientific interest.

Muchandi et al. (50) demonstrated that the ethanolic extract of *P. cubeba* fruits administered to Albino rats protected them against D-galactose induced neuronal lipofuscinogenesis (51). In fact, using a dose of 400 mg/kg, p.o., of *P. cubeba* fruits significantly reduced lipofuscin fluorescence from the hippocampus region of animals comparatively to D-galactose treated rats. In addition, a decrease in the accumulation of lipofuscin granules in hippocampus of animals’ brains was observed in *P. cubeba* treated group. Observed effect was suggested to be due to the richness of the extract in lignans, mainly cubebin, hinokinin, yatein, and isoyatein, that are known as strong antioxidants.

As the intestinal enzymes α-amylase and α-glucosidase are key targets in the regulation of diabetes mellitus, *P. cubeba* extracts were reported as digestive enzymes inhibitors. It was found that the methanolic and aqueous extracts at 1 mg/mL were able to significantly inhibit α-glucosidase and α-amylase *in vitro* (48). Moreover, the anti-diabetes activity of both extracts has been suggested to be likely associated with their antioxidant properties. Next to *P. cubeba*, the root aqueous extract of *P. longum* administered by intraperitoneal route to streptozotocin induced diabetic male Wister albino rats at 200 mg/kg, b.w for 30 days, decreased, by 66.7%, the fasting blood glucose. These studies justify the traditional use of *Piper* species, including *P. cubeba*, and open up promising avenues for the management of diabetes and related complications.

### Antimicrobial activity

#### Antibacterial activity

Nowadays, the increasing prevalence of antibiotic resistance is one of the major challenges for public health worldwide. It has been attributed to the over- and misuse of antibiotics, as well as a declining trend in novel drug development by the pharmaceutical industry and challenging regulatory requirements (52, 53). As plants represent a great resource in drug discovery, for being mostly biocompatible, biodegradable, and less cytotoxic, their extracts and secondary metabolites are being widely explored to discover potential next antimicrobials (29, 54).

Extracts and compounds from *P. cubeba* parts, mainly fruits, were largely evaluated for antimicrobial activity (Table 5). Using four extracts (acetone, hexane, methanol, and ethanol) from *P. cubeba* fruits, Akshita et al. (55) found that all extracts showed high to moderate antibacterial activity against *Klebsiella* sp., *Staphylococcus aureus*, *Escherichia coli*, *Enterococcus* sp., *Enterobacter* sp., and *Pseudomonas aeruginosa* except hexane extract which exhibited no activity (Table 5). The best effects were observed toward *Enterococcus* sp. followed by *E. coli* and *P. aeruginosa* (55). Similar observation was reported that the hexane extract was not active in inhibiting different microbial strains (56). Noteworthy, *P. cubeba* extracts were more effective on Gram-positive than against Gram-negative bacteria. This is due to the single-layer cell wall in Gram-positive strains in contrast to the multilayered cell wall of Gram-negative bacteria that constitutes a barrier for the invasion of antimicrobial agents through the cell membrane.

**TABLE 5:** Antibacterial activity of *P. cubeba.*

Bacteria	Extract	Used method	Effects	References
*E. coli*	Steam-distilled oil	Disk diffusion method	ZI = 9 mm	(111, 112)
*B. subtilis*			ZI = 11 mm	
*V. cholerae*			ZI = 11 mm	
*S. aureus*			ZI = 9 mm	
*S. albus*			ZI = 10 mm	
*S. dysenteriae*			ZI = 10 mm	
*C. dyphteriae*			ZI = 13 mm	
*S. typhi*			ZI = 12 mm	
*S. lutea*			ZI = 9 mm	
*S. faecalis*			ZI = 11 mm	
*B. pumtlus*			ZI = 16 mm	
*S. pyogenes*			ZI = 14 mm	
*M. luteus*			ZI = 8 mm	
*P. solanacearum*			ZI = 30 mm	
*E. coli*	Acetone	Disk diffusion method	ZI = 10 mm; MIC = 0.5 mg/L	(113)
*S. aureus*			ZI = 16 mm; MIC = 1 mg/L	
*P. aeruginosa*			ZI = 13 mm; MIC = 0.5 mg/L	
*E. coli*	Chloroform		ZI = 12 mm; MIC = 0.5 mg/L	
*S. aureus*			ZI = 11 mm; MIC = 1 mg/L	
*P. aeruginosa*			ZI = 10 mm; MIC = 0.5 mg/L	
*E. coli*	Ethanolic		ZI = 10 mm; MIC = 1 mg/L	
*S. aureus*			ZI = 15 mm; MIC = 1 mg/L	
*P. aeruginosa*			ZI = 13 mm; MIC = 0.5 mg/L	
*E. coli*	Aqueous		ZI = 15 mm; MIC = 1 mg/L	
*S. aureus*			ZI = 8 mm; MIC = 1 mg/L	
*P. aeruginosa*			ZI = 15 mm; MIC = 0.5 mg/L	
*B. subtilis*	Essential oil	Disk diffusion method	ZI = 17 mm	(114)
*E. coli* (ATCC43895)	ethanolic extract	Microdilution method	MIC = 0.63 mg/mL; MBC = 1.25 mg/mL	(115)
*B. cereus* (ATCC 11778)	n-hexane	Disk diffusion method	ZI = 12 mm	(105)
	Dichloromethane		ZI = 23 mm	
	MeOH		ZI = 11 mm	
*S. aureus* (NCTC 1803)	n-hexane		ZI = 17 mm	
	Dichloromethane		ZI = 16 mm	
*S. mutans*	Acetone	Agar well diffusion method	ZI = 12.64 mm; MIC = 50 mg/mL	(64)
	Methanol		ZI = 12.31 mm; MIC = 50 mg/mL	
	Ethanol		ZI = 13 mm; MIC = 50 mg/mL	
*S. aureus*	Acetone		ZI = 18.96 mm; MIC = 25 mg/mL	
	Methanol		ZI = 17.65 mm; MIC = 25 mg/mL	
	Ethanol		ZI = 17.32 ± 0.57 mm; MIC = 25 mg/mL	
*B. cereus* (JN 934390)	Essential oil	Agar well diffusion method and microdilution method	ZI = 15.0 mm; MIC = 3.12 mg/mL; MBC = 12.5 mg/mL	(20)
*B. subtilis* (JN 934392)			ZI = 16.0 ± 0.7 mm; MIC = 6.25 mg/mL; MBC = 12.5 mg/mL	
*S. aureus* (ATCC 6538)			ZI = 19.5 mm; MIC = 1.56 mg/mL; MBC = 3.12 mg/mL	
*L. monocytogenes* (ATCC 19115)			ZI = 19.0 mm; MIC = 1.56 mg/mL; MBC = 3.12 mg/mL	
*M. luteus* (NCIMB 8166)			ZI = 16.0 mm; MIC = 1.56 mg/mL; MBC = 6.25 mg/mL	
*K. pneumoniae* (ATCC 10031)			ZI = 13.0 mm; MIC = 3.12 mg/mL; MBC = 6.25 mg/mL	
*S. enterica* (ATCC 43972)			ZI = 23.0 mm; MIC = 12.5 mg/mL; MBC = 25 mg/mL	
*S. typhimurium* (ATCC 19430)			ZI = 13.5 mm; MIC = 6.25 mg/mL; MBC = 12.5 mg/mL	
*E. coli* (ATCC 25922)			ZI = 21.0 mm; MIC = 3.12 mg/mL; MBC = 6.25 mg/mL	
*P. agglomerans*	Essential oil	Agar well diffusion method	ZI = 5 mm	(116)
*X. campestris pv. citri*			ZI = 11 mm	
*S. aureus* MTCC 3103	Diethyl ether oleoresin	Agar well diffusion method	2 μL/well: ZI = 22.mm	(117)
			6 μL/well: ZI = 36.1 mm	
*B. subtilis* MTCC 1790			2 μL/well: ZI = 0 mm	
			6 μL/well: ZI = 13.2 mm	
*E. coli M*TCC 1672			2 μL/well: ZI = 29.2 mm	
			6 μL/well: ZI = 53.3 mm	
*S. typhi* MTCC 733			2 μL/well: ZI = 47.2 mm	
			6 μL/well: ZI = 65.2 mm	
*S. aureus* MTCC 3103	Ethanol oleoresin		2 μL/well: ZI = 12.4 mm	
			6 μL/well: ZI = 18.3 mm	
*B. subtilis* MTCC 1790			2 μL/well: ZI = 18.1 mm	
			6 μL/well: ZI = 32.0 mm	
*E. coli* MTCC 1672			2 μL/well: ZI = 14.3 mm	
			6 μL/well: ZI = 29.3 mm	
*S. typhi* MTCC 733			2 μL/well: ZI = 56.3 mm	
			6 μL/well: ZI = 100 mm	
*S. aureus* MTCC 3103	Petroleum ether, benzene oleoresin		2 μL/well: ZI = 23.6 mm	
			6 μL/well: ZI = 42.1 mm	
*B. subtilis* MTCC 1790			2 μL/well: ZI = 36.3 mm	
			6 μL/well: ZI = 73.3 mm	
*E. coli* MTCC 1672			2 μL/well: ZI = 0 mm	
			6 μL/well: ZI = 21.4 mm	
*S. typhi* MTCC 733			2 μL/well: ZI = 56.4 mm	
			6 μL/well: ZI = 60.0 mm	
*S. aureus* MTCC 3103	Chloroform oleoresin		2 μL/well: ZI = 17.1 mm	
			6 μL/well: ZI = 26.4 mm	
*B. subtilis* MTCC 1790			2 μL/well: ZI = 23.5 mm	
			6 μL/well: ZI = 44.9 mm	
*E. coli* MTCC 1672			2 μL/well: ZI = 50.0 mm	
			6 μL/well: ZI = 68.3 mm	
*S. typhi* MTCC 733			2 μL/well: ZI = 49.2 mm	
			6 μL/well: ZI = 84.1 mm	
*S. aureus* MTCC 3103	Methanol oleoresin		2 μL/well: ZI = 19.2 mm	
			6 μL/well: ZI = 30.2 mm	
*B. subtilis* MTCC 1790			2 μL/well: ZI = 0 mm	
			6 μL/well: ZI = 0 mm	
*E. coli* MTCC 1672			2 μL/well: ZI = 28.5 ± 1.4 mm	
			6 μL/well: ZI = 67.0 ± 0.1 mm	
*S. typhi* MTCC 733			2 μL/well: ZI = 32.6 ± 0.6 mm	
			6 μL/well: ZI = 41.3 ± 2.1 mm	
*S. aureus* MTCC 3103	Essential oil		2 μL/well: ZI = 30.6 ± 0.3 mm	
			6 μL/well: ZI = 50.4 ± 1.6 mm	
*B. subtilis* MTCC 1790			2 μL/well: ZI = 46.6 ± 1.2 mm	
			6 μL/well: ZI = 72.3 ± 1.1 mm	
*E. coli* MTCC 1672			2 μL/well: ZI = 42.0 ± 0.3 mm	
			6 μL/well: ZI = 80.0 ± 0.3 mm	
*S. typhi* MTCC 733			2 μL/well: ZI = 56.3 ± 0.1 mm	
			6 μL/well: ZI = 100	
*E. coli*	Hydro-alcoholic	Disk diffusion method	ZI = 18 ± 0.64 mm	(117)
*S. saprophyticus*			ZI = 19 ± 0.26 mm	
*K. pneumonia*			ZI = 19 ± 0.51 mm	
*P. mirabilis*			ZI = 20 ± 0.41 mm	
*E. coli*		Agar Well method	ZI = 20 ± 0.00 mm	
*S. saprophyticus*			ZI = 18 ± 0.47 mm	
*K. pneumonia*			ZI = 18 ± 0.18 mm	
*P. mirabilis*			ZI = 19 ± 0.37 mm	
*E. coli*	n-Hexane	Agar well diffusion method	ZI = 30 ± 2.3; MIC = 7.5 mg/mL	(117)
*Salmonella* sp.			ZI = 32 ± 2.0; MIC = 5.0 mg/mL	
*S. flexneri*			ZI = 37 ± 1.6; MIC = 5.0 mg/mL	
*V. parahaemolyticus*			ZI = 38 ± 0.6; MIC = 5.0 mg/mL	
*V. cholerae*			ZI = 31 ± 2.0; MIC = 5.0 mg/mL	
*P. aeruginosa*			ZI = 17 ± 0.6; MIC = 5.0 mg/mL	
*L. delbrueckii*			ZI = 25 ± 0.7; MIC = 10.0 mg/mL	
*Brochothrix* sp.			ZI = 11 ± 0.6; MIC = 20.0 mg/mL	
*B. subtilis*	Essential oil	Agar well diffusion method	ZI = 26.5 ± 1.1 mm	(117)
*S. aureus*			ZI = 41.2 ± 1.5 mm	
*B. cereus*			Total inhibition	
*E. coli*			ZI = 25.6 ± 1.5 mm	
*S. typhi*			ZI = 17.0 ± 0.2 mm	
*P. aeruginosa*			ZI = 0 mm	
*B. subtilis*	Tailed pepper oleoresin	Agar well diffusion method	ZI = 32.1 ± 0.8 mm	
*S. aureus*			Total inhibition	
*B. cereus*			ZI = 53.2 ± 1.5 mm	
*E. coli*			ZI = 19.4 ± 1.2 mm	
*S. typhi*			ZI = 33.2 ± 1.1 mm	
*S. aureus*	Acetone	Agar well diffusion method	ZI = 14.0 ± 0.70 mm	
*Klebsiella* sp.			ZI = 14.3 ± 0.18 mm	
*Enterococcus* sp.			ZI = 15.2 ± 0.52 mm	
*P. aeruginosa*			ZI = 15.3 ± 0.62 mm	
*E. coli*			ZI = 16.3 ± 0.75 mm	
*S. aureus*	Methanol	Agar well diffusion method	ZI = 11.5 ± 0.30 mm	
*Enterococcus* sp.			ZI = 17.6 ± 0.80 mm	
*P. aeruginosa*			ZI = 13.2 ± 0.06 mm	
*E. coli*			ZI = 15.0 ± 0.30 mm	
*S. aureus*	Ethanol	Agar well diffusion method	ZI = 9.0 ± 0.05 mm	
*Klebsiella* sp.			ZI = 8.5 ± 0.10 mm	
*Enterococcus* sp.			ZI = 11.3 ± 0.16 mm	
*P. aeruginosa*			ZI = 9.6 ± 0.34 mm	
*E. coli*			ZI = 8.5 ± 0.17 mm	
*S. mutans* KCCM3309	Methanol (100 mg/mL)	Disc Diffusion Assay and microdilution method	ZI = 10.00 mm; MIC = 0.23 mg/mL; MBC = 0.23 mg/mL	(117)
	Ethanol (100 mg/mL)		ZI = 10.33 mm; MIC = 0.10 mg/mL; MBC = 0.10	
	Hexane (100 mg/mL)		ZI = 10.00 mm; MIC = 0.10 mg/mL; MBC = 0.10	
*S. sobrinus* ATCC33478	Methanol (100 mg/mL)		ZI = 12.17 mm; MIC = 0.93 mg/mL; MBC = 16.67 mg/mL	
	Ethanol (100 mg/mL)		ZI = 11.13 mm; MIC = 0.80 mg/mL; MBC = 20.83 mg/mL	
	Hexane (100 mg/mL)		ZI = 12.07 mm; MIC = 0.87 mg/mL; MBC = 20.83 mg/mL	
*A. viscosus* ATCC15987	Methanol (100 mg/mL)		ZI = 11.67 mm; MIC = 0.80 mg/mL; MBC = 20.83 mg/mL	
	Ethanol (100 mg/mL)		ZI = 11.17 mm; MIC = 0.47 mg/mL; MBC = 20.83 mg/mL	
	Hexane (100 mg/mL)		ZI = 12.67 ± 0.58 mm; MIC = 0.87 ± 0.70 mg/mL; MBC = 13.54 ± 10.97 mg/mL	
*S. mutans* KCCM3309	Hexane fraction (100 mg/mL)		ZI = 9.33 mm; MIC = 0.10 mg/mL; MBC = 0.10 mg/mL	
	Ethyl acetate fraction (100 mg/mL)		ZI = 9.66 mm; MIC = 0.13 mg/mL; MBC = 0.13	
	Aqueous methanol fraction (100 mg/mL)		ZI = 11.00 mm; MIC = 0.10 mg/mL; MBC = 0.10	
*S. sobrinus* ATCC33478	Hexane fraction (100 mg/mL)		ZI = 11.13 mm; MIC = 0.67 mg/mL; MBC = 25.00 mg/mL	
	Ethyl acetate fraction (100 mg/mL)		ZI = 11.07 mm; MIC = 3.15 mg/mL; MBC = 25.00 mg/mL	
	Aqueous methanol fraction (100 mg/mL)		ZI = 11.20 mm; MIC = 1.07 mg/mL; MBC = 7.29 mg/mL	
*Ac. viscosus* ATCC15987	Hexane fraction (100 mg/mL)		ZI = 12.00 mm; MIC = 1.58 mg/mL; MBC = 16.67 mg/mL	
	Ethyl acetate fraction (100 mg/mL)		ZI = 10.83 mm; MIC = 1.20 mg/mL; MBC = 25.00 mg/mL	
	Aqueous methanol fraction (100 mg/mL)		ZI = 12.33 mm; MIC = 1.07 μg/mL; MBC = 12.50 μg/mL	

IZ, inhibition zone; MBC, minimum bactericidal concentration; MIC, minimum inhibitory concentration.

Moreover, *P. cubeba* essential oil was also shown to be endowed with good antibacterial activity against methicillin-resistant *S. aureus* ATCC 43300 (Table 5). This was evaluated using atomic force microscopy and transmission electron microscopy. At 50 μg/mL, the essential oil severely damaged the bacterial cell walls while it was not active at microscopic levels at 25 μg/mL. However, at nanoscopic levels, it induced significant perturbation in the bacterial cell wall. These effects on the cell wall and plasma (cytoplasmic) membrane are likely to be the way by which this essential oil impaired bacterial activity (57).

Elsewhere, *P. cubeba* essential oil induced anti-*Helicobacter pylori* activity (MIC = 7.81 μg/mL) and thereby proposed as a therapeutic agent to protect and/or treat *H. pylori* infection (58). *P. cubeba* methanolic extract was also tested as a natural food preservative against microbial population in tofu using total plate counting (TPC) method. A decrease of upper to 3 Log_10_ CFU/g of TPC was observed against *B. cereus*, coliform and *E. coli* in tofu treated with 0.5% of the extract for 4 h. *P. cubeba* L. berries were suitable for use as a natural preservative to reduce the microbial load in raw food (59).

It was suggested that the essential oil targets the cell wall of bacterial cells, whereas the extracts attack and destroy the peptidoglycan causing cell collapse. *P. cubeba* essential oil injures the cell wall−anchored proteins that are involved in biofilm formation and adhesion. It could also injure the cytoplasmic membrane (57). Other *Piper* plants were reported for their huge antibacterial spectrum (60). For instance, *Piper nigrum* L. methanolic and chloroform extracts inhibited *E. coli*, *S. aureus*, *S. typhi*, and *Proteus* sp. except *P. aeruginosa* which was resistant to both extracts (61). Likewise, the leaf ethanolic extract of *Piper betel* L. exhibited pronounced antibacterial activities toward *B. subtilis*, *S. aureus*, *E. coli*, and moderate inhibition of *P. aeruginosa*. The aqueous extract was also tested and was active only against *B. subitilis* (62). These studies corroborate the traditional use of *Piper* plants, including *P. cubeba*, in managing infectious diseases. Therefore, it could serve as a source of novel therapeutic agents against human pathogenic bacteria and food borne pathogens. Alqadeeri et al. (63) isolated and identified, for the first time, two compounds, β-asarone, and asaronaldehyde, from the methanolic extract of *P. cubeba* and its fractions. Both compounds inhibited the growth of *B. pumilus* ATCC14884, *B. cereus* ATCC33019, *B. megaterium* ATCC14581, and *B. subtilis* ATCC6633 (MIC = 63.0–125.0 μg/mL) and inactivated more than 90.99% of the *Bacillus* spores 0.05%. More importantly, the compounds destroyed all the spores at 0.1% after 1 h of incubation. The antibacterial and anti-sporicidal activity of β-asarone and asaronaldehyde provide useful information about the antimicrobial effect of *P. cubeba* (63). However, *in vivo* studies would be of a great importance to support their development as antibacterial agents.

#### Antifungal activity

The antifungal activity of *P. cubeba* was fully explored in many studies by using several methods mainly agar disc diffusion, well diffusion method, microdilution, inverted petri plate, and poison food medium assays (Table 6). The antifungal potential of five extracts of *P. cubeba* berries against the opportunistic oral fungal pathogens *Candida albicans* and *Saccharomyces cerevisiae* was studied using the MIC assay (64). The acetone extract was the most potent against both species followed by the methanolic and ethanolic extracts. *C. albicans* was more sensitive than *S. cerevisiae* (64). Similarly, Salkar et al. (65) developed an oral gel from the essential oil (0.5%) of *P. cubeba* and tested its activity toward different strains of *Candida*. The gel elicited excellent activity against both normal (*C. albicans* ATCC 10231, *C. glabrata* H04FS fluconazole sensitive, *C. krusei* G06FS fluconazole sensitive) and resistant (*C. albicans*-fluconazole resistant, *C. krusei* G03FR fluconazole resistant, *C. glabrata* H05FR fluconazole resistant) *Candida* species. Interestingly, the developed oral gel was endowed with comparable inhibitory effect to marketed local-herbal gel samples (65). Hence, the crude extracts as well as essential oil from *P. cubeba* fruits were considered as promising treatments of oral fungal infections, especially *C. albicans* species.

**TABLE 6:** Antifungal activity of *P. cubeba.*

Fungi	Extract/compound	Used method	Effects	References
*G. lucidum*	Essential oil	Disk diffusion method	ZI = 10 mm	(114)
*C. albicans*	Acetone extract	Agar well	ZI = 15.31 ± 0.57 mm; MIC = 12.5 mg/mL	(64)
	Methanol extract	diffusion	ZI = 12.31 ± 0.57 mm; MIC = 25 mg/mL	
	Ethanol extract	method	ZI = 11.94 ± 1 mm; MIC = 25 mg/mL	
*S. cerevisiae*	Acetone extract		ZI = 10.93 ± 1 mm; MIC = 12.5 mg/mL	
	Methanol extract		ZI = 11.31 ± 0.57 mm; MIC = 12.5 mg/mL	
	Ethanol extract		ZI = 11.64 ± 0.57 mm; MIC = 12.5 mg/mL	
*Pythium catenulatum* (AY598675)	Essential oil	Microdilution method	ZI = 13.0 ± 0.5 mm; MIC = 6.25 mg/mL; MFC = 25 mg/mL	(20)
*Fusarium oxysporum* (AB586994)			ZI = 17.0 ± 0.1 mm; MIC = 3.12 mg/mL; MFC = 12.5 mg/mL	
*Fusarium* sp. (JX391934)			ZI = 15.0 ± 0.8 mm; MIC = 3.12 mg/mL; MFC = 12.5 mg/mL	
*C. albicans* ATCC 10231	Essential oil at 0.5 %	Agar dilution method	MIC = 50 mg/mL	
*Candida albicans*-fluconazole resistant			MIC = 50 mg/mL	
*Candida krusei* G03*^FR^* fluconazole resistant			MIC = 60 mg/mL	
*Candida krusei* G06*^FS^* fluconazole sensitive			MIC = 60 mg/mL	
*Candida glabrata* H04*^FS^* fluconazole sensitive			MIC = 50 mg/mL	
*Candida glabrata* H05*^FR^* fluconazole resistant			MIC = 60 mg/mL	
*G. candidum* (TMa 001)	Ethanol extract of berries	Disc diffusion method	ZI = 7.26 ± 0.20 mm	
*P. citrinum* (GRd 001)			ZI = 7.13 ± 0.20 mm	
*T. hirsuta* (LMd 001)			ZI = 13.80 ± 1.40 mm	
*G. candidum* (TMa 001)	Methanol extract of berries		ZI = 8.10 ± 0.80 mm; MIC = 1.25 mg/mL; MFC = 2.5 mg/mL	
*P. citrinum* (GRd 001)			ZI = 7.67 ± 0.90 mm; MIC = 0.625 mg/mL; MFC = 1.25 mg/mL	
*T. hirsuta* (LMd 001)			ZI = 18.30 ± 3.00 mm; MIC = 0.039 mg/mL; MFC = 0.078 mg/mL	
*G. candidum* (TMa 001)	Methanol extract of berries		Conidial germination = 3.1%	
*P. citrinum* (GRd 001)			Conidial germination = 10.0%	
*T. hirsuta* (LMd 001)			Conidial germination = 21.6%	
*Penicillium purpurogenum* (MTCC 1786)	Diethyl ether	Inverted petri plate method	2 μL: ZI = 28 mm	
			6 μL: ZI = 52 mm	
*Fusarium oxysporum* (MTCC 284)			2 μL: ZI = 18 mm	
			6 μL: ZI = 21 mm	
*Fusarium proliferatum* (MTCC 2935)			2 μL: ZI = 36.2 mm	
			6 μL: ZI = 45 mm	
*Penicillium madriti* (MTCC 3003)			2 μL: ZI = 31.3 mm	
			6 μL: ZI = 35 mm	
*Penicillium purpurogenum* (MTCC 1786)	Ethanol		2 μL: ZI = 13 mm	
			6 μL: ZI = 36 mm	
*Fusarium oxysporum* (MTCC 284)			2 μL: ZI = 10 mm	
			6 μL: ZI = 14 mm	
*Fusarium proliferatum* (MTCC 2935)			2 μL: ZI = 60 mm	
			6 μL: ZI = 75 mm	
*Penicillium madriti* (MTCC 3003)			2 μL: ZI = 62.5 mm	
			6 μL: ZI = 65 mm	
*Penicillium purpurogenum* (MTCC 1786)	Petroleum benzene		2 μL: ZI = 35 mm	
			6 μL: ZI = 69 mm	
*Fusarium oxysporum* (MTCC 284)			2 μL: ZI = 0 mm	
			6 μL: ZI = 2 mm	
*Fusarium proliferatum* (MTCC 2935)			2 μL: ZI = 32 mm	
			6 μL: ZI = 40 mm	
*Penicillium madriti* (MTCC 3003)			2 μL: ZI = 37.5 mm	
			6 μL: ZI = 42 mm	
*Penicillium purpurogenum* (MTCC 1786)	Chloroform		2 μL: ZI = 48 mm	
			6 μL: ZI = 83 mm	
*Fusarium oxysporum* (MTCC 284)			2 μL: ZI = 15 mm	
			6 μL: ZI = 22 mm	
*Fusarium proliferatum* (MTCC 2935)			2 μL: ZI = 13 mm	
			6 μL: ZI = 40 mm	
*Penicillium madriti* (MTCC 3003)			2 μL: ZI = 42 mm	
			6 μL: ZI = 53 mm	
*Penicillium purpurogenum* (MTCC 1786)	Methanol		2 μL: ZI = 30 mm	
			6 μL: ZI = 61 mm	
*Fusarium oxysporum* (MTCC 284)			2 μL: ZI = 3.7 mm	
			6 μL: ZI = 10 mm	
*Fusarium proliferatum* (MTCC 2935)			2 μL: ZI = 8.7 mm	
			6 μL: ZI = 25 mm	
*Penicillium madriti* (MTCC 3003)			2 μL: ZI = 56 mm	
			6 μL: ZI = 62 mm	
*Penicillium purpurogenum* (MTCC 1786)	Essential oil		2 μL: ZI = 90 mm	
			6 μL: ZI = 100 mm	
*Fusarium oxysporum* (MTCC 284)			2 μL: ZI = 12.5 mm	
			6 μL: ZI = 35 mm	
*Fusarium proliferatum* (MTCC 2935)			2 μL: ZI = 10 mm	
			6 μL: ZI = 12.5 mm	
*Penicillium madriti* (MTCC 3003)			2 μL: ZI = 65 mm	
			6 μL: ZI = 73 mm	
*Fusarium proliferatum* (MTCC 2935)			2 μL: ZI = 52 mm	
			6 μL: ZI = 62.3 mm	
*Penicillium madriti* (MTCC 3003)			2 μL: ZI = 41.7 mm	
			6 μL: ZI = 70 mm	
*Trichophyton rubrume*	Fruit 70% Ethanol extract	Microdilution method	MIC = 8 mg/mL	
	Fruit hot-water extract		MIC > 8 mg/mL	
	Terbinafine		MIC = 2 μg/mL	
*Alternaria porri*	Essential oil	Disc diffusion method	IZ = 7 ± 1 mm	
*Fusarium oxysporum* f. sp cicer			IZ = 7.5 ± 1.29 mm	

IZ, inhibition zone; MBC, minimum bactericidal concentration; MIC, minimum inhibitory concentration.

Many oleoresins from *P. cubeba* fruits were tested against different food pathogenic fungi (66) (Table 6). Using inverted petri plate assay, the chloroform oleoresin at 6 μL was highly active against *Penicillium purpurogenum*. However, the petroleum benzene oleoresin was ineffective against *Fusarium oxysporum* at all doses. Other oleoresins elicited minimum to moderate activities. Using the food poison technique, the ethanol oleoresin at 6 μL was effective against *Penicillium madriti*. Many other fungal species were sensitive to *P. cubeba* essential oil such as *Aspergillus fumigatus*, *A. flavus*, and *F. solani*, among others. The antifungal activities of *P. cubeba* extracts and essential oils are presented in Table 6.

Comparatively, the crude methanolic extract and fractions (dichloromethane, hexane, and ethyl acetate) from *Piper solmsianum*, as well as four pure compounds namely eupomatenoid-5, eupomatenoid-3, conocarpan and orientin were all assessed against 12 pathogenic fungi (67). The methanolic extract and fractions elicited a good antifungal effect against all the dermatophytes strains (MIC, μg/mL = 20–60), a weak activity against the zigomycetes and were not active toward the hyaline hyphomycetes. Compounds eupomatenoid-5, conocarpan, and orientin exhibited pronounced activities against all the dermatophytes tested (MIC ≤ 1–9 μg/mL). Noteworthy, conocarpan showed a remarkable activity against all the yeasts. To sum up, the antifungal activity of *P. cubeba* and its relatives seems to be promising and is likely related to the presence of bioactive compounds belonging to neolignanes and flavonoids. However, the presence of other active compounds should be verified and evaluated.

### Antiparasitic and antileishmanial activities

In addition to the antimicrobial activities, *P. cubeba* essential oil was also active against *Schistosoma mansoni*, the trypomastigote and amastigote forms of *Trypanosoma cruzi*, and the promastigote forms of *Leishmania amazonensis* (68). The *in vitro* inhibitory effect against *T. cruzi* was dose dependent. In contrast, essential oil was inactive toward *L. amazonensis*.

*In vivo*, a recent study showed that intraperitoneal treatment of male BALB/c mice by encapsulated and unencapsulated (−)-cubebin isolated from *P. cubeba* showed up to 61.3% reduction in the number of the trypomastigotes of a strain of *T. cruzi*. Animals treated with encapsulated (−)-cubebin survived longer compared to those treated with Benznidazole used as standard antiparasitic drug (69). These findings open a promising application of encapsulated (−)-cubebin as antiparasitic agent.

Other *Piper* species were also reported for antiparasitic purposes. For instance, *P. dennisii* was shown to exhibit anti-plasmodial activity *in vitro* (70). Moreover, benzoic acid derivatives isolated from *P. acutifolia* and *P. glabratum* were effective toward both *T. cruzi* and *Plasmodium falciparum* (71). *In vitro* evaluation of extracts from different *Piper* plants such as *P. barbatum*, *P. aduncum*, *P. acutifolium*, and *P. dilatatum* showed that they are potent in inhibiting *T. cruzi* (72). These findings demonstrate that the antiparasitic potential of *Piper* plants, including *P. cubeba*, is worth exploring in drug discovery.

Antileishmanial activity of *P. cubeba* extracts was evaluated *in vitro* toward *Leishmania donovani* promastigotes (73). All tested extracts (n-hexane, ethyl acetate, methanol, and acetone) elicited a significant activity at 100 μg/mL with more than 90% inhibition. In the case of n-hexane extract, two lignans namely cubebin and hinokinin, were identified and isolated. Cubebin exhibited a significant *in vitro* antileishmanial activity at 100 μM. *In vivo* experiment carried out in golden hamsters against *L. donovani* amastigotes showed that cubebin slightly reduced parasitic burden and spleen weight (73). Comparatively, the antileishmanial activity was also demonstrated by of *P. aduncum* extracts (74). Moreover, the essential oils from *P. angustifolium* were effective against *Leishmania infantum* (75). Also *P. cubeba* exhibited anthelmintic activity against earthworms and tapeworms *in vitro* (76).

### Wound-healing activity

Medicinal plants are the major source of wound healing products with more than 70% while the remaining sources are mineral and animal-based pharma products (77–79). Several plants are known to accelerate wound healing (80). However, only few studies have explored this activity from *P. cubeba*. Shakeel et al. (81) assessed the wound healing effect of *P. cubeba* essential oil using self-nanoemulsifying drug delivery system (SNEDDS). Prepared formulation was evaluated for wound healing, collagen determination, and histo-morphological examination in female Wistar rats. Upon oral administration, it was found that EO-SNEDDS formula significantly accelerated wound healing and enhanced collagen content in tested animals in comparison with pure essential oil. Noteworthy, histopathological evaluation of the formula-treated animals showed no signs of inflammatory cells indicating that it is safe to female rats (81).

More recently, essential oil from *P. cubeba* fruits (PCEO) was tested for *in vivo* wound healing potential (20). Tested PCEO induced a powerful antibacterial activity especially against *Listeria monocytogenes* and *S. aureus*, known to be involved in wound infections. Interestingly, the application of PCEO as topical cream accelerated the wound healing process, increased the SOD level, and reduced the malondialdehyde (MDA) level. In addition, histopathological examination demonstrated that the derma was restored and arranged properly. The observed activities were attributed to the synergy between the antioxidants and antimicrobials present in PCEO.

Phytochemicals seem to elicit wound healing activity by targeting several factors mainly those known to be responsible for delaying and/or reducing the wound healing process such as infections, deficiency in blood supply, diabetes mellitus, necrotic tissue, and lymphatic blockage (82). Within *Piper* genus, the aqueous leaf extract of *P. betle* applied to wounds *in vivo* induced a significant contraction and complete epithelization of the wounds after 10 and 14 days of treatment, respectively (83). Elsewhere, topical application of the ointments prepared from the leaves, stems, and roots of *P. hayneanum* significantly improved the healing of rats’ wounds and reduced the infections by two wound pathogens: *S. aureus* and *C. albicans* (84). In conclusion, it is apparent that *Piper* plants contain active principles with great potential to be used as topical ointments to enhance wound healing and prevent the establishment of wound-related infections.

### Immunomodulatory activity

Phytochemicals, such as terpenoids, polysaccharides, glucosides, flavonoids, and alkaloids, are widely reported as immunomodulators to some extent (85). As for *P. cubeba*, the immunomodulatory activity of its protein extracts was evaluated on the proliferation of immune cells using MTT assay on the splenocytes. This was tested in presence and absence of the mitogenic agent, concanavalin-A (Con-A) (86). The protein extracts exhibited a more significant immunosuppressive activity compared to the total extract. In addition, Ikawati et al. (87) demonstrated that the hexane and ethanolic extracts of *P. cubeba* fruits caused lysis of 2H3 cells leading to the release of high level of histamine (87). This effect was comparable to that induced by the standard drug, Thapsigargin. These results suggest the potential of *P. cubeba* extracts to face allergic diseases. However, *in vivo* experiments would provide further useful information to identify the bioactive molecules and explain the underpinning mechanisms. Using another *Piper* member, the administration of the methanolic extract of *P. longum* and its major principle piperine, induced a significant increase in the total white blood cell (WBC) count, enhanced the bone marrow cellularity, and increased circulating antibody titer, α-esterase positive and plaque forming cells in Balb/c mice (88).

Several other phytochemicals have been evaluated for immunomodulatory purposes and some mechanisms of action have been uncovered. For instance, epigallocatechin-3-gallate (EGCG) was able to inhibit NF-kB activation and down-regulate the production of NO in macrophages as well as the expression of monocyte chemoattractant protein-1 (MCP-1). Moreover, resveratrol, the highest renowned active molecule in grapevine, acted via inhibiting TNF-α and/or (LPS)-mediated macrophages, NF-kB, dendritic cells, and myeloid (89).

### Hepato- and renoprotective activity

As the need for anti-hepatitis C virus (HCV) agents is growing, the search for new candidates that can serve as drugs or as core-entities to design an effective HCV inhibitor and its enzymes is promising. *P. cubeba* aqueous extract inhibited HCV-PR activity *in vitro* with an IC_50_ of 18.0 μg/mL (45). When compared to other plants tested in this study (45), *P. cubeba* aqueous and methanol extracts were among the most active by inducing 94.2 ± 2.1 and 84.7 ± 1.8% inhibition at 100 mg/mL, respectively. In an attempt to study the renoprotective potential of *P. cubeba*, a 47 years old male patient diagnosed with hypertension induced chronic kidney disease (CKD) and altered serum creatinine level which was unable to revert to normal levels using the conventional medication, was orally given two capsules of *P. cubeba* at 4 g/day for 6 weeks (90). This resulted in a significant improvement in subjective symptoms (anorexia and fatigue) as well as the objective parameters of the disorder (blood urea, serum creatinine and urine routine and microscopy). In addition, no adverse effects were observed during and after the study. It was concluded that *P. cubeba* boost the effectiveness in reducing serum creatinine level and in increasing estimated glomerular filtration rate (eGFR) and may help reduce further complications related to renal parenchymal damage.

In another study, the antilithiatic activity of the hydroalcoholic extract of *P. cubeba* fruits was investigated in male Sprague Dawley rats (91). Animals having received the extract showed a significant decrease in crystals level in urine. Moreover, a reduction in serum creatinine and urea was also observed. Interestingly, magnesium in animals’ urine was increased while sodium, calcium, phosphorus, and chloride were significantly decreased. Likewise, histopathological examination showed a clear improvement in kidney tissue in treated rats with *P. cubeba* extract following induction of urolithiasis by ethylene glycol and ammonium chloride. This study strongly suggests that *P. cubeba* could be of significant utility in inhibiting calcium oxalate urolithiasis. Comparatively, streptozotocin induced diabetic Wister albino rats treated by the root aqueous extract of *P. longum* maintained the normal activities of hepatic [serum glutamic oxaloacetic transaminase (SGOT), serum glutamic pyruvic transaminase (SGPT), alkaline phosphatase (ALP)] and renal (serum creatinine and urea) functional markers. This showcases the protective and biosafety roles of *P. longum* extract against diabetes induced liver and kidney damages. In addition, the extract elicited an antihyperlipidemic activity demonstrated by a significant decrease in the total cholesterol (TC), very low density lipoprotein (VLDL), triglycerides (TG), low density lipoprotein (LDL), and an increase in the high density lipoprotein (HDL) (92). Besides, *P. cubeba* essential oil was also investigated for antihyperuricemic activity and showed strong effect against xanthine oxidase (IC_50_ = 54.87 μg/mL) compared to *P. nigrum* EO (IC_50_ = 77.11 μg/mL) (7).

### Melanogenesis activity

The hydroethanolic extract of *P. cubeba* fruits was evaluated for melanogenesis stimulation activity using cultured murine B16 melanoma cells. At 10 mg/ml, the extract enhanced both intracellular and extracellular melanin contents comparatively to the negative control. In contrast, no significant effect was observed on cell proliferation rate. This stimulatory effect on melanin was attributable to the presence of cubebin, a known constituent of *P. cubeba* fruits (93). Comparatively, the extract of *P. methysticum* and *P. nigrum* also showed a strong stimulatory activity on melanogenesis. Following up these findings, guided bioassay allowed the isolation of two kavalactones yangonin and 7,8-epoxyyangonin from *P. methysticum*. When tested, both kavalactones significantly stimulated the melanogenesis in B16 melanoma cells (93). Nevertheless, more in deep studies are recommended to uncover the molecular targets and underpin the mechanisms of action.

### Antidepressant activity

The antidepressant potential of *P. cubeba* EO was investigated *in vivo* using Albino mice and Fluoxetine, a selective serotonin reuptake inhibitor, as antidepressant standard drug (94). Using forced swimming method, animals treated with essential oil gained weight, exhibited more mobility, and showed less immobility comparedatively to the mice treated with Fluoxetine. This reduction in passive behavior in animals highlighted the antidepressant-like effect of *P. cubeba* essential oil. Interestingly, piperine from *P. nigrum* was studied for its antidepressant-like effect using corticosterone-induced model of depression in mice for 3 weeks. Relative to the control animals, those treated with piperine showed a significant decrease in sucrose utilization and an increase in immobility time. In addition, it maintained the levels of brain-derived neurotrophic factor protein and mRNA (95). This demonstrates the antidepressant-like effect of piperine. In another recent study, seven compounds often found in *P. nigrum* (paprazine, pellitorine, piperine, sylvamide, cepharadione A, piperolactam D, and 10-tricosanone) were docked against two receptors namely the potassium channel and human serotonin transporter to assess their action on the anxiolytic and antidepressant activities observed *in vivo*. Results showed that tested compounds interact with these target proteins with docking scores ranging from −1.0 to −7.9 kcal/mol indicating that they are likely responsible for the antidepressant activity (96). Nevertheless, as the antidepressant activity is still poorly explored, further experiments with different *Piper* species, compounds, methods, and *in vivo* models are needed.

### Insecticidal activity

*P. cubeba*, especially its EO, was evaluated for its plant-based insecticides activity (97). It was proved that at 0.003125%, the EO significantly repelled *Sitophilus oryzae* adults. This effect was more potent than those induced by pure compounds α-pinene and β-caryophyllene. Following fumigation, the EO was the most potent in causing lethality of *S. oryzae* adults (LC_50_ = 1.07 mL cm^–3^ air). Comparatively, the EO and α-pinene exhibited more toxic effect compared to *Zingiber officinale* EO and β-caryophyllene. The noticed insecticidal activity was attributed to the ability of the EO to inhibit acetylcholinesterase enzyme (AchE) in fumigated rice weevil (*S. oryzae*). Moreover, the oviposition of *Callosobruchus* sp. was significantly reduced after fumigation with *P. cubeba* EO. Similarly, a combination of 4-methyl-3-heptanol and *P. cubeba* EO was revealed to be more effective as a bait for *Scolytus scolytus* than multilure traps. This was due to the synergetic action between α-cubebene and 4-methyl-3-heptanol (98). Many other *Scolytinae* species including *Xyleborini* and *Corthylini* tribes were presented to be sensitive to *P. cubeba* based compounds such as α-copaene, α-cubebene, α-humulene, and calamenene. Similarly, myristicin (4-methoxy-6[2-propenyl]-1,3-benzodioxole) isolated from the hexane fraction of *P. mullesua* D. Don fruits induced significant toxicity against the 4th instar larvae of *Spilarctia obliqua* after 24 h of topical application (LD _50_ = 104 μg/larva) (99). Additionally, it was showed that in *Piper* genus, piperamides are the major compounds with the strongest insecticidal activity. Many extracts from *P. nigrum*, *P. guineense*, and *P. tuberculatum* were shown to be active against insect pests (100). In conclusion, Piper plants and compounds constitute an innovative source of biopesticide agents for controlling insects out-breaks.

Moreover, *P. cubeba* largely inhibited the germination and growth of tow weeds (*Bidens pilosa* and *Echinochloa crus-galli*). Noteworthy, *P. cubeba* EO reduced photosynthesis in the two weeds while lipid peroxidation electrolyte leakages were increased at 1.93 mg/mL (7).

## Biological activities of isolated compounds

Nature is the storehouse of many active compounds that we are currently using as pharmaceuticals. *P. cubeba* synthesizes many secondary metabolites, among them hinokinin, cubebin and cubebin derivatives that are reported to be the most pharmacologically active compounds. These compounds exhibited many biological activities mainly antimicrobial, anticancer, antimutagenic, antiparasitic, ovicidal, and anticholinesterase (Table 7). In fact, lignans from *P. cubeba* were shown to alter the expression of PTGS2 and MMP2 proteins in head and neck cancer cells (12). Additionally, (−)-cubebin derivatives, (−)-hinokinin, and (−)-*O*-benzyl cubebin (OBZ) at 40 mg/kg inhibited the inflammation *in vivo* induced by injection of either PGE2 or dextran into the paw of animals in comparison to indomethacin, the reference standard (101). Besides these activities, (−)-cubebin was found to exert a vasorelaxant effect mediated by the NO/cGMP signaling pathway without prostacyclin participation (102). In another study, sixteen compounds were isolated from *P. cubeba* extracts based on their antioxidant potential to scavenge free radicals, hydroxyl radical, superoxide anion radical, and DPPH (103). It was mainly found that crotepoxide was the most active against 5,5-dimethyl-1-pyrroline-N-oxide-OH with up to 57% inhibition. In contrast, less inhibitory activity was noticed using other compounds such as 1′-acetoxychavicol acetate, deoxypipoxide and 3-(3′,2′,5′ -trimethoxyphenyl) pyrrolidine. Moreover, several compounds including 5,6-dehydrokawain, benzyl benzoate, 1′-acetoxychavicol acetate, deoxypipoxide, and 5,7,3′,4′-tetrametoxyflavone exhibited superoxide dismutase (SOD)-like activity through their ability to deliver protons (103).

**TABLE 7:** Biological activities of the isolated compounds from *P. cubeba.*

Compound name	Bioactivity	Results	References
(−)-Cubebin	MIC assay against *E.*	MIC = 0.20–0.35 mM	(118)
(−)-Hinokinin	*faecalis, S. salivarius, S. sanguinis, S. mitis, S. mutans, S. sobrinus, C. albicans*	MIC = 0.25–0.32 mM	
(−)-O-(N,N-dimethylaminoethyl)-cubebin		MIC = 0.19–0.31 mM	
(−)-*O*-Benzyl cubebin		MIC = 0.18–0.31 mM	
(−)-6,6’-Dinitrohinokinin		MIC = 0.18–0.30 mM	
5-methoxy-yatein	Antiparasitic activity against *Schistosoma mansoni* worms	At 10–100 μM, 100% of parasites presented a motor activity decrease	(119)
(−)-Hinoquinin; (−)-cubebin; yatein		Motor activity was decreased by 75 and 100% at concentrations of 50 and 100 μM, respectively	
(−)-Cubebin	Cytotoxicity, mutagenicity, and expression of p38 MAP kinase and GSTa2 in a hepatoma cell line	HTC cells exposed to 28 mM of (−)-cubebin for 24 h did not show altered expression of p38 MAP kinase and GSTa2.	(118)
(−)-Cubebin	*In vitro* anticancer activity against A549 (human lung adenocarcinoma), K562 (human chronic myeloid leukemia), SiHa (human cervical carcinoma), and HCT116 (human colon carcinoma) cell lines using MTT assay	IC_50_ = 8.30–45.2 μM	(120)
(−)-Dihydrocubebin		IC_50_ = 7.82–85.32 μM	
Cyclic ether cubebin		IC_50_ = 7.94–73.88 μM	
(−)-Hinokinin		IC_50_ = 7.86–72.58 μM	
Amide derivatives of (−)-cubebin		IC_50_ = 6.61–93.51 μM	
Succinimide derivatives of (−)-cubebin		IC_50_ = 7.36–71.8 μM	
Cubebin	Anticancer activity against head and neck cancer cell lines (the larynx (Hep-2) and oral (SCC-25) squamous cell carcinoma cells) using MTT assay	Significant decreased in the proliferation, migration, and genotoxic profile of tested cell lines at concentrations ranging from 10 to 50 μg/mL.	(12)
Methylcubebin			
Cubebin	Ovicidal activity against gastrointestinal nematodes in sheep	EC_50_ = 150.00 μg/mL	(121)
Dihydrocubebin		EC_50_ = 186.70 μg/mL	
Hinokinin		EC_50_ = 68.38 μg/mL	
Cubebin	Larval development test (LDT)	EC_50_ = 14.89 μg/mL	
Dihydrocubebin		EC_50_ = 30.75 μg/mL	
Hinokinin		EC_50_ = Not determined	
Cubebin	L3 migration inhibition test (LMT)	EC_50_ = 0.89 μg/mL	
Dihydrocubebin		EC_50_ = Not determined	
Hinokinin		EC_50_ = 0.34 μg/mL	
(−)-*O*-Methylcubebin, (−)-O-benzylcubebin	MIC assay	MIC = 50 g/ml against *P. gingivalis*	(122)
(−)-Hinokinin		MIC = 100 g/ml against *B. fragilis*	
(−)-Hinokinin	Antimutagenic activity	Animals treated with different doses of (−)-hinokinin (10, 20, and 40 mg/kg b.w.) showed no genotoxic effect and reduced chromosome damage induced by doxorubicin.	(123)
Cubebin	Anticholinesterase activity	IC_50_ = 992 μM	(124)
β-Asarone and asaronaldehyde	MIC assay against *Bacillus* sp.	ZI = 7.21–9.61 mm; MIC = 63.0–125.0 μg/mL; MBC = 250.0–500.0 μg/mL	(63)
Crotepoxide	Radical scavenging activity	% Inhibition at 1.25 mmol/L = 56%	(103)

## General discussion

The present review comprehensively summarized the available literature on the uses of *P. cubeba* and its phytochemicals to promote health conditions and manage diseases-related issues. It also critically addressed the opportunity of using the plant as a source of natural drugs in clinical trials. *P. cubeba* has edible fruits and condiments with various medicinal properties. The most dominant phytochemicals characterized in *P. cubeba* were polyphenolics and flavonoids (rutin, catechin, gallic acid, caffeic acid, ferulic acid, etc.), lignans (Cubebininolide, hinokinin, yatein, and isoyatein, etc.), fatty acids (lauric acid, hexadecanoic acid, palmitic acid, 9-octadecenoic acid, etc.), and volatile compounds (eugenol, β-cubebene, α-cubebene). Owing to this phytochemical richness, the plant has proved a large spectrum of biological and pharmacological activities that corroborate the traditional uses. Moreover, *P. cubeba* volatiles and aromatic characteristics are used in cosmetics for deodorants production and in food industry as culinary flavor (9).

Regarding the medical applications, cubeb’s different extracts and compounds have demonstrated biosafety status both *in vitro* and *in vivo.* In addition, due to the presence of high amounts of polyphenols, *P. cubeba* extracts/compounds exhibited substantial antioxidant/scavenging and anti-inflammatory activities. This is mainly targeted by downregulating the expression of proinflammatory transcriptional factors and cytokines while augmenting the enzymatic and non-enzymatic antioxidants (45). These latter are in turn involved in maintaining hepatic and renal functional parameters. Interestingly, many research investigations were devoted to the anticancer potential of the plant. The toxicity of different phytochemicals against tumor cell lines is mainly due their capacity to suppress many pro-oncogenic pathways and genes and to stimulate tumor suppressor-like pathways (125). Moreover, elicitation of proapoptotic proteins and impairment of mitochondrial membrane potential could also be targeted by plant compounds (78). The plant phytochemicals were also corroborated to be useful in managing diabetes because of their inhibitory effect on diabetic intestinal enzymes such as α-amylase and α-glucosidase (48) as well as in fighting microbial infections by inhibiting pathogens’ growth and quorum sensing (57). Thanks to the antimicrobial, antioxidant, and immunomodulatory activities it induces, *P. cubeba* was also capable of accelerating wound healing process by enhancing blood supply, synthesizing collagen and inhibiting wound infections (20, 82). Overall, *C. cubeba*’s activities, like those elicited by most plants, occur through the modification of the metabolism or gene expression modification.

## Conclusion and outlook

The present work has systematically and comprehensively reviewed the botany, traditional uses, phytochemistry and pharmacology of *P. cubeba* extracts and constituents. In recent years, an increasing interest was given to this plant as it is used in traditional medicine in many countries. Most of these traditional uses have been validated by pharmacological studies. Nevertheless, there is no yet systemic data regarding the pharmacokinetics and clinical research of *P. cubeba*. Therefore, there is not enough evidence to interpret the specific mechanisms for the observed biological activities. Also, there are a few studies to date on other parts of *P. cubeba* than the fruits. To ensure full utilization of the plant, it is necessary to investigate the chemical constituents of each part and tissue. According to current investigations, lignans are the main active constituents of the plant, in which cubebin is the most abundant. This lignan is known to possess several activities like anti-inflammatory, anticancer, analgesic, and antimicrobial. Next to it, other cubebin derivatives were also isolated from the fruits of the plant. Thus, it will be more interesting to investigate the biological activities of the isolated compounds from each part of the plant. This review also highlighted the need to study the bioavailability of cubebin, as it is the major compound, in terms of water solubility and bioavailability *in vivo* following different doses and modes of administration. In addition, more toxicity studies as well as preclinical trials are required using cell-based and animal models. Active extracts warrant establishing guided bioassay experiments to translate the beneficial effects into solid scientific data that can lead to molecules and/or formulas with a targeted therapeutic potential. Another therapeutic strategy could be combining *cubeba*’s major compounds to standard drugs as adjuvants. Finally, despite the continued progress on various aspects of *P. cubeba*, the elaboration and discovery of new drugs from it will need more advanced trials in preclinical and clinical phases.

## Author contributions

BD, IM, MY, and WB reviewed the literature and wrote the manuscript. LB and MS revised the manuscript, designed, and conceived the work. All authors approved the final version.

## Footnotes

1
http://www.worldfloraonline.org
